# Liver Growth Factor (LGF) Upregulates Frataxin Protein Expression and Reduces Oxidative Stress in Friedreich’s Ataxia Transgenic Mice

**DOI:** 10.3390/ijms17122066

**Published:** 2016-12-09

**Authors:** Lucía Calatrava-Ferreras, Rafael Gonzalo-Gobernado, Diana Reimers, Antonio S. Herranz, María J. Casarejos, Adriano Jiménez-Escrig, Javier Regadera, Juan Velasco-Martín, Manuela Vallejo-Muñoz, Juan José Díaz-Gil, Eulalia Bazán

**Affiliations:** 1 Service of Neurobiology, Ramón y Cajal Institute for Health Research (IRYCIS), 28034 Madrid, Spain; luciacalatrava@gmail.com (L.C.-F.); rgonzalo-ibis@us.es (R.G.-G.); diana.reimers@hrc.es (D.R.); antonio.sanchez@hrc.es (A.S.H.); m.jose.casarejos@hrc.es (M.J.C.); manuela.vmqm@gmail.com (M.V.-M.); 2 Service of Neurology, Ramón y Cajal Hospital, 28034 Madrid, Spain; jimeneze@yahoo.com; 3 Departamento de Anatomía, Histología y Neurociencia Facultad de Medicina Universidad Autónoma de Madrid, 28400 Madrid, Spain; javier.regadera@uam.es (J.R.); juan.velascomartin@uam.es (J.V.-M.)

**Keywords:** liver growth factor, Friedreich’s ataxia, neuroprotection, oxidative stress, frataxin

## Abstract

Friedreich’s ataxia (FA) is a severe disorder with autosomal recessive inheritance that is caused by the abnormal expansion of GAA repeat in intron 1 of FRDA gen. This alteration leads to a partial silencing of frataxin transcription, causing a multisystem disorder disease that includes neurological and non-neurological damage. Recent studies have proven the effectiveness of neurotrophic factors in a number of neurodegenerative diseases. Therefore, we intend to determine if liver growth factor (LGF), which has a demonstrated antioxidant and neuroprotective capability, could be a useful therapy for FA. To investigate the potential therapeutic activity of LGF we used transgenic mice of the FXNtm1MknTg (FXN)YG8Pook strain. In these mice, intraperitoneal administration of LGF (1.6 μg/mouse) exerted a neuroprotective effect on neurons of the lumbar spinal cord and improved cardiac hypertrophy. Both events could be the consequence of the increment in frataxin expression induced by LGF in spinal cord (1.34-fold) and heart (1.2-fold). LGF also upregulated by 2.6-fold mitochondrial chain complex IV expression in spinal cord, while in skeletal muscle it reduced the relation oxidized glutathione/reduced glutathione. Since LGF partially restores motor coordination, we propose LGF as a novel factor that may be useful in the treatment of FA.

## 1. Introduction

Cerebellar ataxias (CA) include a group of diseases characterized by a lack of motor coordination mainly related with dysfunction of the cerebellum and associated neuronal circuits. Friedreich ataxia (FA) is a severe disorder with autosomal recessive inheritance that is caused by the abnormal expansion of GAA repeat in intron 1 of FRDA gen. This alteration leads to a partial silencing of transcription of frataxin (FXN), its encoded protein, causing a multisystem disorder disease that includes neurological damage, mainly spinocerebellar ataxia, and non-neurological signs such as hypertrophic cardiomyopathy and diabetes Mellitus type 2. The degree of reduction in FXN expression tightly correlates with the severity of the disease [1,2]. Although FXN’s exact function remains unknown, available evidence supports a role in iron metabolism. The major cellular consequences of its deficiency include: impairment of iron-sulfur clusters biogenesis, altered cellular iron metabolism with cytosolic iron depletion and mitochondrial iron accumulation, increased oxidative stress, and mitochondrial dysfunction [3,4]. Several therapeutic strategies have expanded rapidly over recent years [5]. Because the success of these therapies for FA is limited [6], there is a need to search for new molecules with beneficial effects on damaged organs in order to ameliorate the quality of life of FA patients.

Liver growth factor (LGF) is a 64 kDa molecule purified in 1986 by Díaz-Gil and colleagues from the plasma of partially hepactomized rats [7]. They demonstrated that LGF is an albumin–bilirubin complex, the concentration of which is nearly undetectable in sera from healthy humans or rats, but dramatically increases in the presence of hepatobiliary disorders or liver injury [8,9]. Recent studies show that LGF is a pleiotropic factor capable of stimulating cell proliferation, and tissue regeneration in hepatic and extrahepatic pathologies [10,11,12,13,14]. The use of LGF as a neural tissue regenerator has been recently protected (Patent No US 2014/8,642,551 B2) [15]. The administration of LGF stimulates the sprouting of DA terminals in the striatum of hemiparkinsonian rats [16,17,18], stimulates the generation of new neurons, and promotes their migration in this experimental model of Parkinson’s disease [17], and its delivery into the brain stimulates the survival of grafted fetal neural stem cells and their differentiation to an endothelial-like phenotype [19].

Several studies revealed that LGF modulates the functional homeostasis through its anti-inflammatory and antioxidant action. Thus, in spontaneously hypertensive rats (SHR) LGF improved nitric oxide availability through the reduction of superoxide anion levels, and normalized the plasmatic levels of several oxidative stress markers [20]. LGF also exerted a considerable antioxidant activity in vitro due to its effectiveness as a scavenger of several reactive oxygen species involved in cardiovascular disorders [21]. In addition, this factor reduced the expression of proinflammatory proteins in hemiparkinsonian rats [16] and protected calbindin-positive terminals from 3-acetylpiridine neurotoxicity by reducing the extracellular glutamate concentration and the activation of microglia in the cerebellum [22].

Considering the antioxidant activity of LGF [20,21], as well as the positive effects produced by the factor in a rat model of CA [22], we decided to analyze the therapeutic properties of this growth factor in an FA experimental model [transgenic mice of the FXNtm1MknTg (FXN) YG8Pook strain]. The results showed that peripheral administration of LGF exerts a neuroprotective effect on sensory neurons located in the lumbar spinal cord and reduced cardiac hypertrophy. Both events may be due to increased expression of FXN promoted by the factor in the spinal cord and the heart, since neurodegeneration in both structures is directly related to the protein deficiency. Interestingly, LGF stimulates AKT phosphorylation in the spinal cord, and increases Bcl2/Bax ratio in the brain stem and cerebellum. Furthermore, LGF also reduced oxidative stress in skeletal muscle and improved motor coordination in this experimental model of FA.

## 2. Results

### 2.1. Liver Growth Factor (LGF) Ameliorates Motor Coordination in YG8R Mice

**Figure 1:**
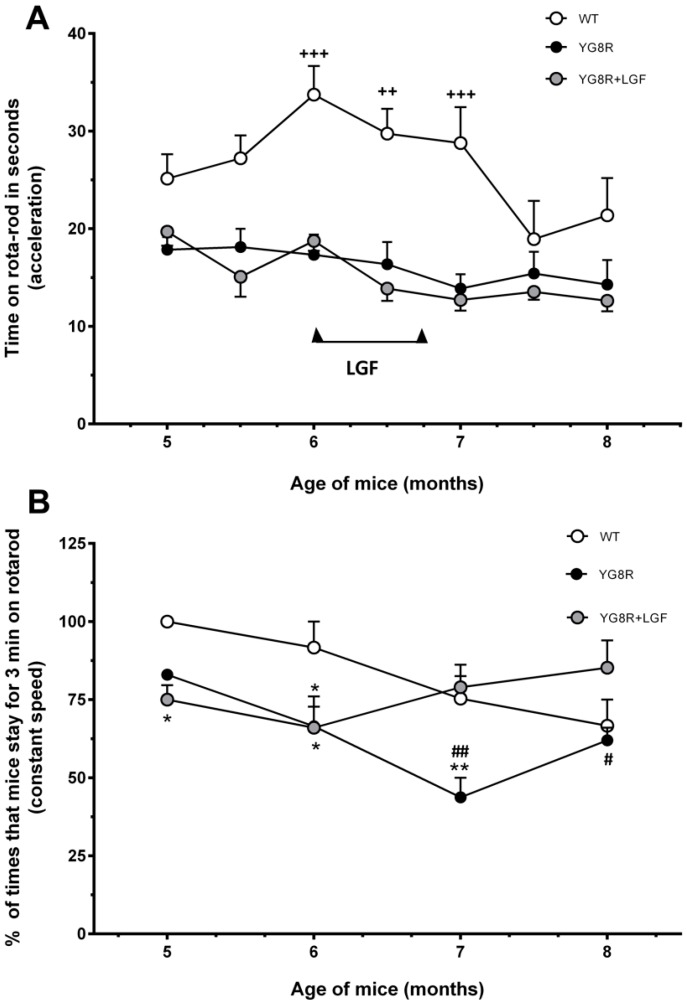
Liver growth factor (LGF) treatment partially restores motor coordination in YG8R mice. (**A**) Shows the time (in seconds) spent on the rotating rod at increasing acceleration of the three groups of animals observed, demonstrating that under this demanding rotarod condition no beneficial effect of LGF is detected; In (**B**) the % of times that mice stay on the rotating rod for 3 min at constant speed (4 rpm) in the WT (**white** circles), YG8R (**black** circles), or YG8R+LGF (**grey** circles) mice. Note the poorer performance of YG8R versus WT mice of five to seven months, and that shortly after treatment values are significantly higher in the LGF-treated than in the vehicle-treated YG8R mice. Results are the mean ± Standard Error of Mean (SEM) of 6–8 independent animals. ^++^ *p* < 0.01, ^+++^ *p* < 0.001 vs. YG8R; * *p* < 0.05, ** *p* < 0.01 vs. WT; ^#^ *p* < 0.05, ^##^ *p* < 0.01 vs. YG8R+LGF.

The effects of LGF IP treatment on motor abilities and coordination were studied using the Rota Rod test. As shown in Figure 1A, the motor performance of wild-type (WT) mice showed a progressive impairment that reached a plateau between seven and eight months of age. By contrast, in YG8R and YG8R+LGF mice motor performance was stable between five and eight months of age, and significantly reduced at six to seven months, as compared with WT mice (Figure 1A).

**Figure 2:**
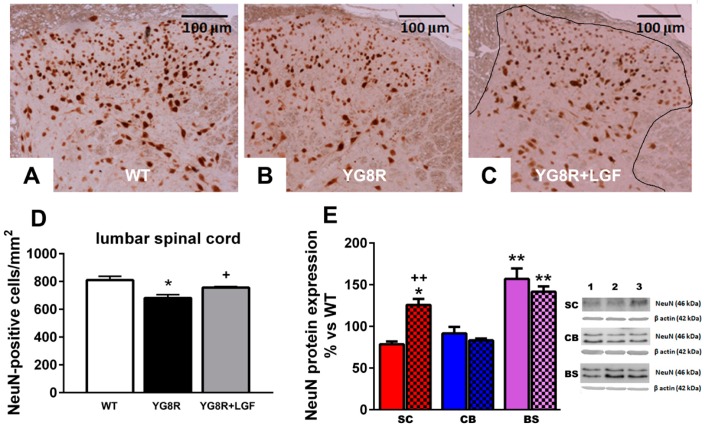
LGF treatment exerts a neuroprotective effect on neurons of lumbar spinal cord in YG8R mice. The upper panel depicts neurons of dorsal horn (**black** delimited area in (**C**)) stained for NeuN (**brown**) in the lumbar spinal cord of WT (**A**), YG8R (**B**), and YG8R+LGF mice (**C**); (**D**) represents the quantification of these neurons, revealing a significant reduction of neuronal loss in YG8R mice treated with LGF (**gray** bar) versus YG8R mice treated with vehicle (**black** bar); (**E**) shows the effect of LGF treatment on NeuN expression, detected by western blot in the spinal cord (SC, **red** bars), cerebellum (CB, **blue** bars), and brain stem (BS, **lilac** bars) of YG8R (**uniform** bars) and YG8R+LGF (bars with **mesh**). Note that LGF significantly increases NeuN expression in spinal cord, whereas it has no effect in other brain regions. **Lane 1** (WT), **Lane 2** (YG8R), **Lane 3** (YG8R+LGF). Results are the mean ± SEM of three (in the case of the spinal cord) or six (in the rest of structures) independent animals. * *p* < 0.05, ** *p* < 0.01 vs. WT; ^+^ *p* < 0.05, ^++^ *p* <0.01 vs. YG8R.

When we analyzed motor performance at constant speed (4 rpm), we observed that at five to seven months of age the percentage of times that YG8R mice stayed for three minutes in rotarod was lower than in WT mice (Figure 1B). At seven to eight months, YG8R+LGF mice showed a significant increase in this parameter, as compared with YG8R mice (Figure 2B), suggesting that LGF treatment improved motor coordination in these mice.

### 2.2. LGF Prevents Neuronal Lost in Spinal Cord of YG8R Mice

Motor performance impairment could be due to the presence of damaged neurons in the dorsal root ganglia and/or spinal cord. Apparently, no changes were observed in the distribution and expression of NeuN in the lumbar spinal cord of 8–9-month-old YG8R mice (Figure 2A–C). However, the total number of NeuN-positive neurons was significantly reduced in those mice compared with WT and YG8R+LGF mice (Figure 2D).

We also analyzed NeuN protein expression in the spinal cord, brain stem, and cerebellum. As shown in Figure 2E, NeuN protein levels were slightly reduced in the spinal cord of YG8R mice, and LGF treatment significantly increased this parameter above that observed in WT mice. In the brain stem of YG8R and YG8R+LGF mice NeuN levels were upregulated, as compared with WT mice, while this protein was not affected in the cerebellum (Figure 2E).

Other studies have described the presence of big vacuoles in the dorsal root ganglia of six- to 12-month-old YG8R mice [23], but under our experimental conditions we were unable to note this alteration.

**Figure 3:**
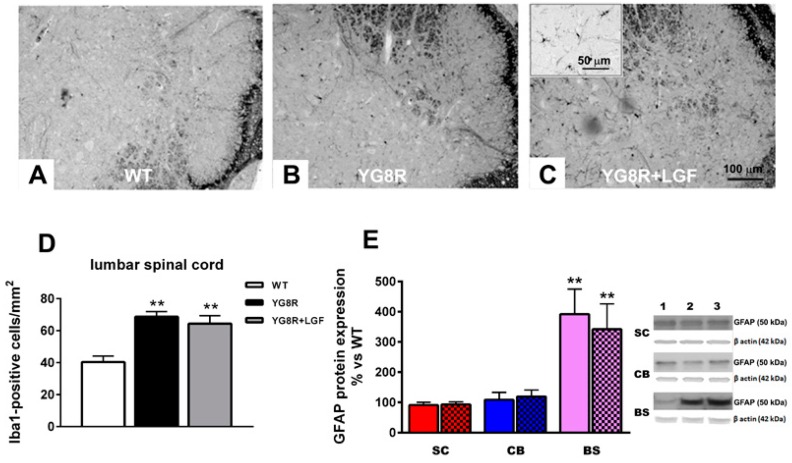
Effect of LGF treatment in microglia and Glial Fibrillary Acidic Protein (GFAP) protein expression. The upper panel depicts activated microglia stained for Iba1 (**brown**) in the lumbar spinal cord of WT (**A**), YG8R (**B**), and YG8R+LGF mice (**C**); The inset in C shows Iba1-positive cells at higher magnification; (**D**) Represents the quantification of these cells, revealing a significant increase in YG8R (**black** bar) and YG8R+LGF mice (**gray** bar) versus WT (**white** bar); (**E**) Shows the effect of LGF treatment on GFAP expression, detected by western blot in the spinal cord (SC, **red** bars), cerebellum (CB, **blue** bars), and brain stem (BS, **lilac** bars) of YG8R (**uniform** bars) and YG8R+LGF (bars with **mesh**). Note how GFAP levels are upregulated in the BS of YG8R and YG8R+LGF mice. **Lane 1** (WT), **Lane 2** (YG8R), **Lane 3** (YG8R+LGF). Results are the mean ± SEM of three (in the case of the spinal cord) or six (in the rest of structures) independent animals. ** *p* < 0.01 vs. WT.

Activated microglia are an important source of oxidative stress that could contribute to the etiopathology of FA. As shown in Figure 3A–D, a significantly higher number of Iba1-positive activated microglia was observed in the spinal cord of YG8R and YG8R+LGF mice, as compared with WT mice. Other studies in FA patients have proposed that neuronal degeneration could be due to the effect on the Schwann cells and satellite glia. We did not observe any alteration in S-100 (specific marker of Schwann cells) and Glial Fibrillary Acidic Protein (GFAP) immunoreactivity in the dorsal root ganglia of YG8R mice, and GFAP protein expression was not affected in the spinal cord of these mice, compared with WT mice (Figure 3E). However, GFAP protein expression was raised in the brain stem of YG8R mice, but LGF did not prevent this increase (Figure 3E).

### 2.3. LGF Treatment Reverses Cardiac Hypertrophy inYG8R Mice

**Figure 4:**
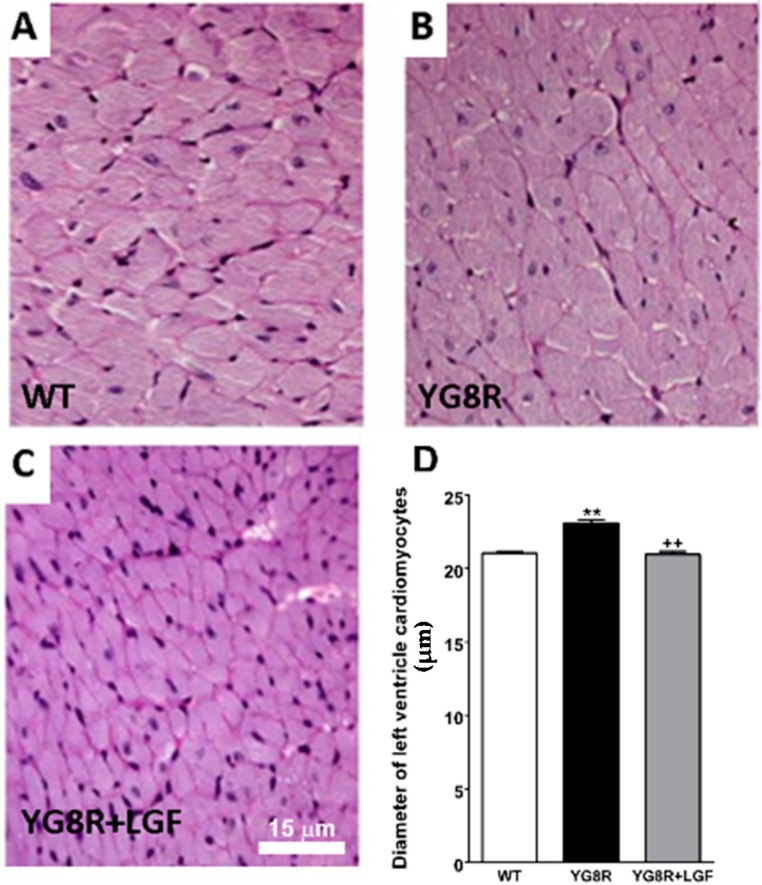
LGF treatment ameliorates cardiac hypertrophy in YG8R mice. (**A**) to (**C**) show hematoxylin (**blue**) and eosin staining (**pink**) of left ventricle coronal sections of WT, YG8R, and YG8R+LGF mice, Scale bar = 15 µm; (**D**) Represents the quantification of the diameter of cardiomyocytes in the three experimental groups. Note the increase of this parameter in YG8R mice (**black** bar), which is prevented with LGF treatment (**gray** bar). Results in (**D**) are the mean ± SEM of 360–420 cardiomyocytes from six independent mice in each experimental group. ** *p* < 0.01 vs. WT; ^++^ *p* < 0.01 vs. YG8R.

Cardiac hypertrophy is observed in an important number of FA patients. As shown in Figure 4, the diameter of left ventricle cardiomyocytes in YG8R mice was significantly higher than in WT mice. This effect was not observed in YG8G+LGF mice, suggesting that LGF treatment reduces cardiac hypertrophy in this experimental model of FA. However, LGF treatment did not affect the total number of cardiomyocytes in the left ventricle. Thus, no differences were observed in this parameter between the different experimental groups of mice analyzed in this study (465 ± 121, 477 ± 128, 427 ± 108 cardiomyocytes nuclei/mm^2^ in WT, YG8R and YG8R+LGF mice, respectively. *n* = 6 independent mice in each experimental group).

### 2.4. LGF Treatment Modulates Frataxin Expression in YG8R Mice

**Figure 5:**
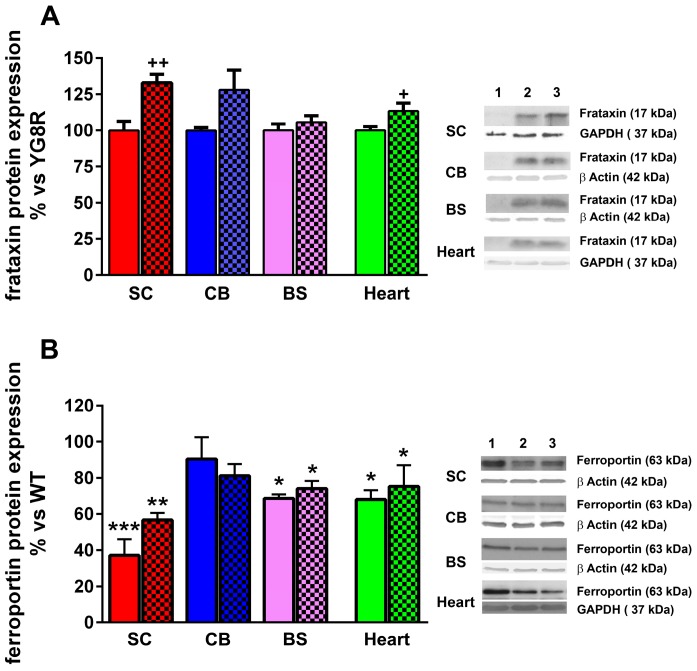
LGF treatment increases frataxin expression in YG8R mice spinal cord. (**A**) represents the effect of LGF on frataxin expression, analyzed by Western blot in the spinal cord (SC, **red** bars), cerebellum (CB, **blue** bars), brain stem (BS, **lilac** bars), and heart (**green** bars) of WT, YG8R (**uniform** bars), and YG8R+LGF (bars with **mes**h) mice. Note that LGF enhances frataxin expression in the spinal cord and heart; In (**B**) the effect of LGF on ferroportin expression is observed in the same structures. Note that the level of this compound is significantly reduced in most structures in YG8R mice versus WT mice. **Lane 1** (WT), **Lane 2** (YG8R), **Lane 3** (YG8R+LGF). Results are the mean ± SEM of three (in the case of spinal cords) or 6–8 (in the rest of structures) independent animals. * *p* < 0.05, ** *p* < 0.01, *** *p* < 0.001 vs. WT; ^+^ *p* < 0.05, ^++^ *p* < 0.01 vs. YG8R.

Frataxin is a mitochondrial protein involved in cellular iron use and maintenance of the redox status which expression is reduced in FA. By using an antibody that recognizes human FXN we observed that LGF treatment increased FXN protein expression by 1.3- and 1.2-fold in the spinal cord and heart of YG8R mice, respectively (Figure 5A). However, no significant changes in FXN levels were observed in the cerebellum and brain stem of these mice.

Ferroportin (FPN) is another protein involved in iron transport that is affected in FA [24]. FPN protein expression was downregulated in the spinal cord, brain stem, and heart of eight- to nine-month-old YGR8 mice, as compared with WT mice, and LGF treatment was unable to recover FNP levels to control values in none of these structures (Figure 5B).

### 2.5. Effect of LGF Treatment in the Expression of Proteins Involved in Cell Survival

**Figure 6:**
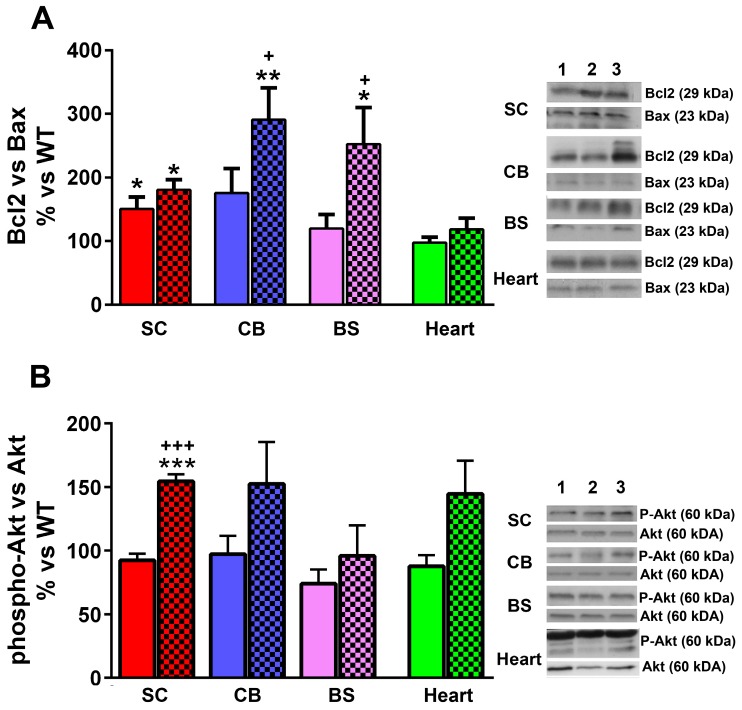
LGF treatment increases the expression of molecules involved in cellular survival in several structures of YG8R mice. (**A**) The effect of LGF on the ratio of Bcl2/Bax expression, analyzed through western blot in the spinal cord (SC, **red** bars), cerebellum (CB, **blue** bars), brain stem (BS, **lilac** bars), and heart (**green** bars) of WT, YG8R (**uniform** bars), and YG8R+LGF (bars with **mesh**) mice. Note that LGF exerts an anti-apoptotic effect in the cerebellum and brain stem; (**B**) The effect of LGF on the ratio of P-Akt/Akt. Note that LGF treatment significantly increases the ratio in the spinal cord and heart. P-Akt, a mediator in the PI3K/Akt signaling pathway, is essential in the regulation of neuronal survival. **Lane 1** (WT), **Lane 2** (YG8R), **Lane 3** (YG8R+LGF). Results are the mean ± SEM of three (in the case of spinal cord) or 6–8 (in the rest of structures) independent animals. * *p* < 0.05, ** *p* < 0.01 vs. WT; *** *p* < 0.001 vs. WT; ^+^ *p* < 0.05, ^+++^ *p* < 0.001 vs. YG8R.

In our previous studies we proposed that the neuroprotective activity of LGF is mediated by the regulation of proteins that are critical for cell survival such as Bcl2 and Akt [16,22]. As shown in Figure 6A, the Bcl-2/Bax ratio was significantly higher in the spinal cord of YG8R than in WT mice. LGF treatment did not modulate this ratio in this structure, but significantly raised it in the cerebellum and brain stem (Figure 6A). On the other hand, we observed that the ratio phospho-Akt/Akt was significantly raised in the spinal cord of YG8R+LGF mice, as compared with WT and YG8R mice. A similar result was observed in the cerebellum and heart of these mice, but this effect was not statistically significant in these structures (Figure 6B).

### 2.6. LGF Treatment Modulates Cytochrome C and Complex IV Protein Expression and Reduces Oxidative Stress in YG8R Mice

**Figure 7:**
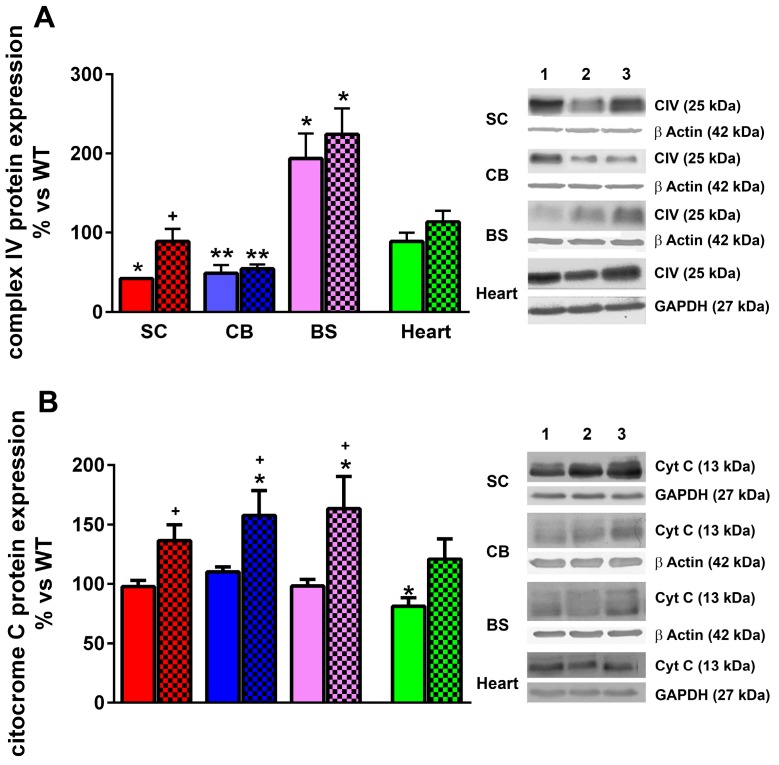
LGF treatment modulates Complex IV and cytochrome C expression in YG8R mice. (**A**) The effect of LGF on Complex IV (CIV) expression, analyzed by western blot in the spinal cord (SC, **red** bars), cerebellum (CB, **blue** bars), brain stem (BS, **lilac** bars), and heart (**green** bars) of WT, YG8R (**uniform** bars), and YG8R+LGF (bars with **mesh**) mice. Note that the level of this compound is significantly reduced in the spinal cord and cerebellum in YG8R mice versus WT mice, and LGF only reverses CIV reduction in the spinal cord; In (**B**) the effect of LGF on cytochrome C is observed in the same structures. Note how cytochrome C expression is reduced in the heart of YG8R mice, and how LGF treatment increases its levels in all the structures analyzed. **Lane 1** (WT), **Lane 2** (YG8R), **Lane 3** (YG8R+LGF). Results are the mean ± SEM of three (in the case of spinal cords) or 6–8 (in the rest of structures) independent animals. * *p* < 0.05, ** *p* < 0.01 vs. WT; ^+^ *p* < 0.05 vs. YG8R.

Complex IV of the mitochondrial respiratory chain catalyzes the transference of electrons from cytochrome C to O_2_ to produce water. A reduction in its activity was reported in lymphoblasts from FA patients [25]. As shown in Figure 7A, complex IV protein levels were significantly reduced in the spinal cord and cerebellum of eight-month-old YG8R mice, while this protein was overexpressed in their brain stem. LGF treatment restored complex IV levels to control values in the spinal cord without affecting the expression of this protein in the other brain structures analyzed (Figure 7A). Although cytochrome C levels are also reduced in FA cells [25,26], only the heart of YG8R mice showed a significant decrease in its expression, as compared with WT mice (Figure 7B). The administration of LGF restored cytochrome C levels to control values in the heart, and upregulated its expression in the spinal cord, cerebellum, and brain stem (Figure 7B).

**Figure 8:**
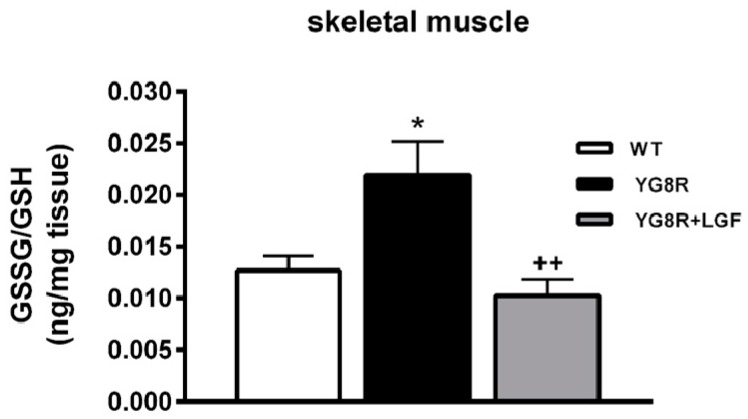
LGF treatment decreases oxidative stress in YG8R mice skeletal muscle. The figure represents the ratio of oxidized glutathione (GSSG) to reduced glutathione (GSH) in the skeletal muscle of WT (**white** bar), YG8R (**black** bar), and YG8R+LGF (**gray** bar) mice. Note that the ratio is significantly increased in YG8R mice versus WT mice, while LGF treatment restores this alteration. Results are the mean ± SEM of 6–8 independent animals. * *p* < 0.05 vs. WT; ^++^ *p* < 0.01 vs. YG8R.

Glutathione is the most abundant non-enzymatic antioxidant in cells [27] and its oxidized form is increased in FA [25,28]. In the skeletal muscle of YG8R mice, the ratio of oxidized glutathione (GSSH) to reduced glutathione (GSH) was upregulated. LGF treatment reduced this parameter to the values observed in WT mice (Figure 8). This effect was due to the increase in GSH promoted by the factor in this structure (313 ± 54, 263 ± 46, +449 ± 69 ng/g of tissue in WT, YG8R and YG8R+LGF mice, respectively. ^+^ *p* < 0.05 vs. YG8R, *n* = 6 independent mice in each experimental group).

## 3. Discussion

In the experimental model of FA used in this study (YG8R mouse), LGF treatment prevented neuronal loss and reduction of Complex IV expression in association with an increase in Frataxin and in P-Akt/Akt ratio in the spinal cord; it increased Bcl2/Bax ratio in the brain stem and cerebellum; it reduced hypertrophy in the heart; and it reduced the ratio of oxidized glutathione versus reduced glutathione in skeletal muscle. LGF’s beneficial effects correlate with a partial restoration of motor coordination in YG8R+LGF mice.

The YG8R transgenic mice is considered a good model of FA that shows a similar, but milder, neuropathology to that presented in FA patients [23]. Thus, the presence of big vacuoles in the neurons of the dorsal root ganglia of the lumbar spinal cord of these mice clearly indicates their degeneration. Although we were unable to detect these vacuoles at any of the different levels of the spinal cord analyzed (lumbar, thoracic, and cervical), we observed an important reduction in the number of NeuN-positive neurons in the spinal cord of eight-month-old YG8R. To our knowledge, this is the first study that shows neuronal depletion in YG8R mice, especially in an area (lumbar spinal cord dorsal horn) where the sensitive neurons that project to the cerebellum through the spinocerebellar tract, and that are affected in FA, are located [29]. According to several studies in FA patients, neuronal degeneration could be due to the affectation of the Schwann cells and satellite glia [30,31]. We did not observe any alteration in S-100 and GFAP immunoreactivity in the dorsal root ganglia of YG8R mice, and GFAP protein expression was not affected in the spinal cord of these mice, compared with WT mice. However, in this latter structure the presence of Iba1-positive activated microglia was increased by 1.7-fold, suggesting that chronic inflammation could be responsible for the reduction of neurons observed in the spinal cord of YG8R.

Neuronal loss was not observed in the lumbar spinal cord of YG8R+LGF mice, and LGF treatment upregulated NeuN protein expression in this structure. Apparently, this neuroprotective effect was not due to a reduction in microglia Iba1-positive, but we cannot exclude a potential anti-inflammatory effect due to the factor. In fact, long-term LGF treatment reduced the expression of some proinflammatory associated proteins (TNF-α and OX6) in hemiparkinsonian and ataxic rats [16,22]. On the other hand, in an experimental model of CA we reported that LGF neuroprotection could be mediated through the modulation of the anti-apoptotic protein Bcl2 [22]. As shown here, LGF treatment did not modulate the Bcl2/Bax ratio in the spinal cord of YG8R mice, suggesting that the overexpression of Bcl2 does not mediate the neuroprotective effect of LGF observed in this structure. However, our results suggest that LGF could modulate the activity of the PI3K/Akt signaling pathway, because the ratio phospho-Akt/Akt was upregulated in the spinal cord of IG8R+LGF mice. This signaling pathway plays a critical role in the regulation of neuronal survival, and, as reported in an experimental model of Parkinson´s disease [16], it could be partially involved in the neuroprotective activity of LGF observed in this study. Our results also show that LGF upregulates Bcl2/Bax ratio in the cerebellum and brain stem of YG8R mice, indicating a potential neuroprotective effect of the factor in these brain structures that are affected in FA patients.

Hypertrophic cardiomyopathy is the first cause of mortality in FA patients. Their heart is enlarged and shows interstitial cardiomyopathy, necrosis, and granular degeneration and hypertrophy of fibers [32,33]. Several alterations associated with cardiac hypertrophy have been described in YG8R mice [23]. Here, we show that the size of the left ventricle cardiomyocytes was significantly higher in eight-month-old YG8R than in WT mice. Because the number of cardiomyocytes was similar in both experimental groups, these results are indicative of cardiac hypertrophy in the YG8R mice. In an experimental model of hypertension, chronic administration of LGF improved vascular function [14] and ventricular hypertrophy [10]. Interestingly, in the YG8R mice LGF treatment reduced the size of the cardiomyocytes to a value similar to that observed in WT mice, suggesting that LGF treatment prevents cardiac hypertrophy in this experimental model of FA.

The pathophysiology of FA is due to the reduced amount of frataxin in targeted neural and non-neural cells and tissues. Under our experimental conditions, LGF treatment upregulated frataxin protein levels in the spinal cord and heart of eight- to nine-month-old YG8R mice. In the case of the heart, this increase was significant but very mild. Perhaps this increase would be more evident if we had analyzed the mitochondrial fraction, but we did not perform a subcellular fractionation in this study due to the small amount of tissue obtained from each mouse. The mechanism used by LGF to potentiate frataxin expression is unknown at present, but previous studies pointed out its ability to potentiate protein synthesis in the damaged liver [34]. In addition, LGF is a potent antioxidant [20,35], and a recent report demonstrated that IGF-I also has antioxidant activity and stimulates frataxin expression in neurons and astrocytes deficient of this protein through the Akt/mTOR signaling pathway [36]. As mentioned above, LGF significantly increases the phospho-Akt/Akt ratio in the spinal cord. In the heart of YG8R+LGF mice this ratio was raised by 1.5-fold, so we may suggest that Akt could also participate in the upregulation of frataxin reported in our study.

A recent study has proposed that in FA iron overload could be more cytosolic than mitochondrial [37]. This overload could be due to an increase in iron uptake, or a reduction in its release through cytosolic transporters such as ferroportin. To our knowledge, this is the first study that shows a decrease in ferroportin protein levels in the spinal cord, brain stem, and heart of YG8R mice, which is consistent with the reduction in ferroportin immunoreactivity reported in FA patients [30]. LGF treatment did not prevent this reduction, suggesting that this factor is unable to reverse the cell toxicity induced by cytosolic iron overload in these structures.

Reduced levels of frataxin induce deregulation in the activity of I, II, and III mitochondrial chain complexes [2,38,39,40]. Other studies also described disturbances in complex IV activity in frataxin-deficient cells and lymphoblasts of FA patients [25,41]. Here, we report a reduction in complex IV protein expression in the spinal cord and cerebellum of YG8R mice. Because this mitochondrial complex catalyzes the transference of electrons from cytochrome C to O_2_ to produce water, its reduction could promote an inadequate transfer of electrons from complex III, and an increase in free radicals that probably induces cellular damage. LGF treatment restored complex IV levels and raised cytochrome C protein expression in the spinal cord. The upregulation of both proteins could favor a reduction in the concentration of free radicals in neurons, which could explain the beneficial effects observed in this study. In the cerebellum LGF was unable to upregulate complex IV expression, but increased cytochrome C levels, suggesting a potential compensatory mechanism induced by the factor to regulate complex IV activity in this structure. We should mention that complex IV levels were significantly increased in the brain stem of YG8R mice, and LGF did not modulate its expression. Similar results were observed in NeuN and GFAP levels, so we may consider that the overexpression in this structure of these three proteins is not a previously reported characteristic of the YG8R mice. As observed in the spinal cord and cerebellum, LGF treatment raised cytochrome C expression, suggesting a potential neuroprotective activity of the factor in the brain stem. The heart of the YG8R mice showed a significant decrease in cytochrome C levels that could be involved in a reduction of complex IV activity. LGF probably compensates for the lack of activity in this structure by preventing the reduction in cytochrome C levels, as observed in the heart of YG8R+LGF mice.

Dysregulation of glutathione homeostasis and alterations in glutathione-dependent enzyme activities contributes to the initiation and progression of neurodegenerative diseases including FA [42]. Here we show that the ratio GSSH/GSH was upregulated in the skeletal muscle of YG8R mice, indicating an increase in oxidative stress that, as reported in FA [43], may contribute to cell damage in this structure. A similar result was observed in the dorsal root ganglia of these mice [28], and in frataxin-depleted cells where impairment of glutathione homeostasis and changes in glutathionylation and function of specific proteins were found [25,28,41,44,45,46]. A previous study shows the ability of LGF to reduce oxidative stress in the cardiovascular system of hypertensive rats [21]. We also found a reduction in this parameter because upregulation of the GSSH/GSH ratio was not observed in the skeletal muscle of YG8R+LGF mice. This positive effect was due to an increase in the reduced form of glutathione (GSH) that is critical for protecting tissues from oxidative stress, acting as a free radical scavenger and inhibitor of lipid peroxidation. On the other hand, LGF administration could reduce oxidative stress through the upregulation of the nuclear factor erythroid 2-related factor 2 (Nrf2), as reported in an experimental model of cigarette smoke-induced emphysema [47]. This transcription factor, which is defective in FA patients [43], restores redox homeostasis in the cell by inducing the expression of antioxidant enzymes. Further experiments must be conducted to determine if LGF modulates Nrf2 expression in YG8R and in frataxin-deficient cells obtained from FA patients.

We evaluated the effectiveness of LGF treatment in motor coordination as analyzed by the rotarod. As mentioned above, the YG8R mice partially reproduce the human symptoms of FA. Between three and six months of age, these mice show mild motor coordination failures and a decreased motor activity, and develop ataxia at 24 months [23]. In our hands, failures in motor coordination were only detected at five to seven months in YG8R mice due to the impairment in this parameter observed in eight-month-old WT mice. LGF treatment did not ameliorate motor coordination in accelerating rotarod test. However, at a constant speed (4 rpm) the percentage of times that seven- to eight-month-old YG8R+LGF mice stayed in the rotarod for more than 3 min was higher than in YG8R mice. Perhaps the lack of effect observed in the accelerating test could be due to the demanding parameters used in our study (4 to 40 rpm in 1 min). In fact, other authors had to change their protocol in order to detect an improvement in motor coordination of YG8R mice treated with IGF-I [36]. Thus, although our results suggest that LGF ameliorates motor coordination in YG8R mice, the effectiveness of the factor has to be ascertained by using other parameters in the acceleration test such as those described for IGF-I (4 to 40 rpm in 3 to 5 min).

Finally, from our results we are not able to define if the effects of LGF observed here are due to direct or indirect action of the growth factor on the affected nervous tissue. A direct action would imply that LGF could cross the blood–brain barrier through several mechanisms involved in the transendothelial transport of albumin and albumin-conjugated nutrients [48,49]. Alternatively, LGF could bind to specific receptors present in the blood–brain barrier. The receptors for the advanced glycation end products (RAGE) located in the endothelium could be good candidates, since AGE products have similar biochemical properties to LGF, i.e., they are mostly albumins bound to glucose, which changes albumin conformation [50]. Moreover, RAGE stimulation elicits the release of TNF-α, which is a cytokine that participates in LGF-mediated proliferation and neuroregeneration [15].

## 4. Experimental Section

### 4.1. LGF Purification

LGF was obtained with the highest purity from rat serum following a previously reported procedure [11]. The absence of contaminants and/or other growth factors (purity) in the LGF preparation was also evaluated following the standard criteria described by Díaz-Gil et al. [7,8,9,11]. All LGF preparations showed a single band in SDS-PAGE electrophoresis and were lyophilized and kept at 4 °C until use. Finally, LGF aliquots were dissolved in saline prior to administration.

### 4.2. Ethics Statement

The Ethics Committee of the Hospital Ramón y Cajal, Madrid (animal facilities ES280790002001) approved all the protocols related to the use of laboratory animals. All procedures associated with animal experiments were in accordance with Spanish legislation (RD 53/2013) and the European Union Council Directive (2010/63/EU).

### 4.3. Experimental Model of Friedreich’s Ataxia in Mice

Twelve two- to three-month-old male Fxn ^tm1Mkn^/Tg(FXN)YG8Pook (YG8R) mice were obtained from the Jackson Laboratory (Bar Harbor, ME, USA), and used in accordance with the European Union Council Directive (86/609/EEC). These mice are homozygous for the *Fxn^tm1Mkn^* (frataxin) targeted allele and hemizygous for the *Tg*(*FXN*)*YG8Pook* (frataxin, human) transgene, and show a similar, but milder, neuropathology to that presented in FA patients [23]. As wild-type mice (WT) we used the C57BL/6J strain that was grown in our animal facilities.

### 4.4. Behavioral Testing

Motor performance was analyzed using the rotarod test (PanLab S.L., Mod. LE 8500, Cornellá, Spain). Briefly, mice received four 3-min evaluations at 4 rpm (constant speed), and four 1-min evaluations at 4 to 40 rpm (accelerating rod) once a month, starting at three months of age until the end of the study period. Data at constant speed were expressed as the percentage of times that mice stayed for 3 min on the rotarod at 4 rpm. This percentage was obtained from four independent evaluations/mouse at each experimental time analyzed in this study.

### 4.5. LGF Administration

At six months of age, mice received two weekly IP injections of saline (YG8R, *n* = 6) or LGF (1.7 μg/mice) (YGR+LGF, *n* = 6) for three weeks. This optimal dose of LGF has been used in different model systems using an identical or similar schedule. Mice were sacrificed four weeks after the last treatment with vehicle or LGF. A total of six WT mice were also used as controls.

### 4.6. Tissue Processing

Eight-month-old mice were beheaded under deep anesthesia with isoflurane, and their brain stems, cerebellums, and spinal cords were removed and dissected following a previously described methodology [51]. For histological studies, the spinal cord was isolated from the vertebral column, fixed in 4% paraformaldehyde for four days, and included in HCl + EDTA for 2 h. After several washings the spinal cords were dehydrated and included in paraffin. For histological studies, and since we were interested in analyzing dorsal root ganglia and fibers and ventral root fibers, spinal cords were maintained within their vertebral columns during fixation in 4% paraformaldehyde for four days, and were coronally cut into three pieces corresponding to the cervical, thoracic, and lumbar-sacral cord. Afterwards, vertebral columns were decalcified through treatment with HCl + EDTA for 2 h and, after several washings, spinal cord pieces were dehydrated and included in paraffin.

### 4.7. Antibodies and Immunochemicals (Histology)

The primary antibodies used in this study were: mouse anti-neuronal nuclei (NeuN, 1:1000; Chemicon International Inc., Temecula, CA, USA), mouse anti-frataxin (1:100; Millipore Ibérica S.A.U., Madrid, Spain), and mouse anti-Iba1 (1:300; Wako Chemicals GmbH, Neuss, Germany). The immunochemicals and secondary antibodies that were used in histology are listed below: Alexa Fluor-568 goat anti-mouse IgG, and Alexa Fluor-488 goat anti-rabbit IgG (1:400; all from Molecular Probes; Eugene, OR, USA), diaminobenzidine (DAB) + substrate-chromogen system (both from Dako Cytomation, Carpinteria, CA, USA), streptavidin–biotin–peroxidase complex (Dako Cytomation) and biotinylated goat anti-mouse IgG (Zymed Laboratories, San Francisco, CA, USA).

### 4.8. Immunohistochemistry and Morphometric Analysis

For pathological and immunohistochemical analyses of the spinal cord, 8 μm thick coronal sections from the three medullar levels (lumbar, thoracic, and cervical) were obtained in a microtome (Microm, Mod. HM 325, Walldorf, Germany), and mounted in positive charged slides (DakoCytomation, Copenhagen, Denmark). Tissue sections were treated with sodium acetate 10 mM, pH 6.0, at 95 °C for 4 min, and preincubated with 5% normal goat serum (NGS) in Tris-buffered saline (TBS: 0.15 M NaCl and 0.1 M Tris HCl, pH 7.4)/0.1% Triton X-100 for 30 min. Primary antibodies were applied for 24 h at 4 °C, and most of them were visualized using immunofluorescence procedures. The slides were coverslipped in a medium containing *p*-phenylenediamine and bisbenzimide (Hoechst 33342; Sigma Aldrich, St. Louis, MO, USA) for detection of nuclei.

For pathological studies, sections stained for hematoxylin and eosin from each spinal cord piece (cervical, thoracic, and lumbar) were selected, so that per piece at least two to three dorsal root ganglia and their corresponding spinal cord section were analyzed.

For quantitative estimation of NeuN and Iba1 immunostaining, sections were selected from spinal cord levels where the ventral horn had the widest extension: C6 and C7 of cervical cord pieces, T3 of thoracic cord pieces, and L4 of lumbar cord pieces. The number of immunoreactive cells was quantified in the whole surface of spinal cord grey matter (including both dorsal and ventral horns), which was visualized through images from stitched widefields of each side of the spinal cord, using the 20× lens and Nikon NIS elements software (scan large image function, Nikon, Tokyo, Japan), connected to a Nikon ECLIPSE Ti-e microscope (Nikon), equipped with a Nikon DS-2MV camera. The quantitative estimation was expressed as the number of immunoreactive cells per mm^2^.

### 4.9. Heart Histological Analysis

For histological studies, ventricular tissues were fixed in 4% buffered formalin and embedded in paraffin wax. Tissues were sectioned at 5 µm and stained with hematoxylin-eosin, Masson’s trychrome, and periodic acid–Schiff stain (PAS). Coronal sections of ventricular tissue were taken. Due to randomized histological cut sections stained with Masson’s trychrome, the first subvalvular coronal sample were measured on a millimeter scale (KODAK RFS 3570 Film Scanner, Flubacher + Co., F+C). Data were expressed as surface of muscular ventricular heart per millimeter (mm) of epicardial layer, because all histological sections are not always at exactly the same height and ventricular cavities are different in each sample. The cardiomyocyte size of the left ventricle was measured through photographs of three high-power fields (objective ×40). The minor diameter of cardiomyocytes was measured. Those with a diameter lower than 16 micrometers were rejected for consideration as a dichotomy branch of cells; and those with a diameter higher than 36 micrometers were also rejected because the borders between cardiomyocytes were not well delimited.

### 4.10. Western Blotting Protein Analysis

Heart and brain tissue samples were homogenized (1:8 (*w*/*v*)) using lysis buffer (20 mM Tris-HCl, pH 7.5: 1 mM dithiothreitol, 2 mM benzamidine,0.5% Triton X-100, 10 µg/mL pepstatin A, 140 mM potassium chloride; 5 mM magnesium acetate; 1 mM EDTA, 2 mM EGTA, 10 µg/mL leupeptin 20 mM sodium β-glycerophosphate; 20 mM sodium molybdate, 200 mM sodium orthovanadate, 10 µg/mL antipain, and 2 mM EGTA) and then were centrifuged at 11,000× *g* for 20 min. All procedures were performed at 4 °C. Samples were aliquoted and then kept at −80 °C until used. Protein concentrations were determined for each sample. Samples containing 30 µg of protein were analyzed by SDS-PAGE and transferred onto PVDF membranes (GE Healthcare, Barcelona, Spain). Membranes were blocked with 0.1 M PBS and 5% dry skimmed milk, pH 7.4, and incubated overnight at 4 °C with the primary antibodies mouse anti-NeuN (1:1000; Chemicon International Inc., Temecula, CA, USA), rabbit anti-Akt (^Ser473^P) (1:2000, Cell Signaling Technology, Beverly, MA, USA), rabbit anti-Akt (1:2000, Cell Signaling Technology), rabbit anti-ferroportin (1:1000, Bioss Chemicals, Pamiers, France), mouse anti-frataxin (1:1000, Millipore, Temecula, CA, USA), mouse anti-complex IV (1:1000, Invitrogen, Carlsbad, CA, USA), mouse anti-cytochrome C (1:1000, BD Pharmigen, Haryana, India), and rabbit anti-GFAP (1:500; Dako Cytomation, Denmark), mouse anti-Bcl-2 (1:400; Santa Cruz Biotechnology Inc., Burlingame, CA, USA) and rabbit anti-Bax (1:300; Santa Cruz Biotechnology Inc.). After extensive washing in 0.05% PBS-Tween, membranes were incubated with the peroxidase-conjugated or alkaline-phosphatase-conjugated secondary antibodies diluted 1:2000 in blocking solution. The membranes were developed with enhanced chemiluminescence Western blotting, following the manufacturer’s instructions (Amersham, Buckinghamshire, UK), and were exposed to hyperfilm. Membranes were also immunolabeled for loading control using mouse anti-β actin (1:5000; Sigma Aldrich) or mouse anti-GAPDH (1:5000; Sigma Aldrich) and anti-mouse IgG alkaline phosphatase-conjugated (1:3000, Sigma Aldrich) and were developed with alkaline phosphatase reagent. The density of the stained bands was scanned and quantified with the Image QuantTL software package (GE Healthcare Bio-Sciences AB, Uppsala, Sweden) and the data were normalized in relation to β actin or GAPDH levels.

### 4.11. Glutathione Determination

Total GSH levels were measured following the method described by Tietze [52]. Briefly, tissue from skeletal muscle was lysed in 100 µL of 0.4 N perchloric acid (PCA) for 30 min at 4 °C, and centrifuged. The supernatants were neutralized with four volumes of 0.1 M NaH_2_PO_4_, 5 mM EDTA, pH 7.5. GSH content was measured in a p96 automatic reader by the addition of 5,5-dithio-bis-2-nitrobenzoic acid (DTNB, 0.6 mM), nicotinamide adenine dinucleotide phosphate reduced tetrasodium salt (NADPH, 0.2 mM), and glutathione reductase (1 U). The reaction was monitored at 412 nm for 6 min.

### 4.12. Data Analysis

Results are expressed as mean ± Standard Error of Mean (SEM) of (*n*) independent animals. Statistical analyses for immunohistochemical and biochemical studies were performed using one-way ANOVA followed by the Newman–Keuls multiple comparison test. For behavioral studies, a two-way ANOVA followed by Newman–Keuls multiple comparison test was used. Differences were considered significant when *p* ≤ 0.05.

## 5. Conclusions

1. Among the regions of the central nervous system of eight-month-old YG8R mice analyzed in the present study, the spinal cord displays the most alterations in the parameters evaluated here. This structure shows a significant decrease in the number of neurons in the lumbar region, and in the expression of ferroportin and Complex IV (cytochrome oxidase), while the number of microglia increases.

2. Abnormalities found in other regions include a decrease in Complex IV in cerebellum, hypertrophy in cardiomyocytes, a decrease in ferroportin in the heart, and an increase in the ratio of oxidized glutathione versus reduced glutathione in skeletal muscle.

3. These pathologic changes correlate with the impairment in motor coordination observed in five- to seven-month-old YG8R mice, as compared with WT mice.

4. LGF treatment ameliorates the pathology of YG8R mice through the neurotrophic, antioxidant, and antiapoptotic activities of the factor, and the induction of neuronal and mitochondrial protein expression. Thus, LGF prevents neuronal loss and reduction of Complex IV expression in association with an increase in Frataxin and in the P-Akt/Akt ratio in the spinal cord; it increases Bcl2/Bax ratio in the brain stem and cerebellum; it reduces hypertrophy and increases Frataxin in the heart; and it reduces the ratio of oxidized glutathione to reduced glutathione in skeletal muscle.

5. LGF’s beneficial effects correlate with a partial restoration of motor coordination in YG8R+LGF mice, detectable as a significant increase in the percentage of times that mice stayed for 3 min on the rotating rod at constant speed.
